# Associations between Exposure to Intimate Partner Violence, Armed Conflict, and Probable PTSD among Women in Rural Côte d’Ivoire

**DOI:** 10.1371/journal.pone.0096300

**Published:** 2014-05-13

**Authors:** Jhumka Gupta, Kathryn L. Falb, Hannah Carliner, Mazeda Hossain, Denise Kpebo, Jeannie Annan

**Affiliations:** 1 Division of Social and Behavioral Sciences, Yale School of Public Health, New Haven, Connecticut, United States of America; 2 Department of Chronic Disease Epidemiology, Yale School of Public Health, New Haven, Connecticut, United States of America; 3 Center for Interdisciplinary Research on AIDS, Yale University, New Haven, Connecticut, United States of America; 4 Social and Behavioral Sciences, Harvard School of Public Health, Boston, Massachusetts, United States of America; 5 Gender Violence and Health Centre, London School of Hygiene and Tropical Medicine, London, England; 6 Innovations for Poverty Action, Abidjan, Côte d’Ivoire; 7 International Rescue Committee, New York, New York, United States of America; Massachusetts General Hospital, United States of America

## Abstract

**Background:**

Objectives were to assess associations between intimate partner violence (IPV), violence during armed conflict (i.e. crisis violence), and probable post-traumatic stress disorder (PTSD).

**Methods:**

Using a sample of 950 women in rural Côte d’Ivoire, logistic generalized estimating equations assessed associations between IPV and crisis violence exposures with past-week probable PTSD.

**Results:**

Over one in 5 (23.4%) women reported past-year IPV, and over one in 4 women (26.5%) reported experiencing IPV prior to the past year (i.e. remote IPV). Crisis violence was experienced by 72.6% of women. In adjusted models including demographics, crisis violence (overall and specific forms), and IPV (remote and past-year), women who reported past-year IPV had 3.1 times the odds of reporting probable past-week PTSD (95%CI: 1.8–5.3) and those who reported remote IPV had 1.6 times the odds (95%CI: 0.9–2.7). Violent exposures during the crisis were not significantly associated with probable PTSD (any crisis violence: aOR: 1.04 (0.7–1.5); displacement: aOR: 0.9 (95%CI: 0.5–1.7); family victimization during crisis: aOR: 1.1 (95%CI: 0.8–1.7); personal victimization during crisis: aOR: 1.7 (95%CI: 0.7–3.7)).

**Conclusion:**

Past-year IPV was more strongly associated with past-week probable PTSD than remote IPV and violence directly related to the crisis. IPV must be considered within humanitarian mental health and psychosocial programming.

## Introduction

The mental health and well-being of populations impacted by war has been a critical area of inquiry, with extensive research documenting high frequencies of depressive, anxiety, and psychotic disorders in such settings [1]. Among the numerous mental health concerns that have been documented in settings impacted by conflict, the prevalence of post-traumatic stress disorder (PTSD) has been shown to be consistently high, with lifetime prevalence ranging from 15.8% in Ethiopia to 37.4% in Algeria among randomly sampled survivors of war or mass conflict [2]. In conflicts with especially high levels of civilian targeting and sexual violence, PTSD prevalence may be even higher, with population-based cluster samples reporting a prevalence of 50.1% probable PTSD in the eastern Democratic Republic of Congo [3] and 74.3% probable PTSD in northern Uganda [4]. Though estimates of PTSD in post-conflict West African populations, including Côte d’Ivoire, are less available, the few existing estimates suggest that probable PTSD is highly prevalent with ranges from 10% to 50% [5], [6].

Specific attention to the mental health of women among war-affected populations is warranted as they may be at increased risk for developing PTSD compared to men [7]–[9]. One critical factor to investigate is the mental health impacts of intimate partner violence (IPV) among women in settings impacted by conflict. High frequencies of IPV have been documented in such settings (9.6%–51.7%) [10]–[13], and studies with women in populations not impacted by conflict have shown a greater likelihood of PTSD and other poor mental health outcomes among women with experiences of IPV [14], [15].

A growing collection of studies among war-affected youth (e.g. Afghani and Palestinian) have indicated that family-related and or interpersonal violence are more robust predictors of poor mental health than war-related violence [16], [17]. Similar to war-affected youth, consideration of these multiple forms of violence is important for research regarding the mental health of adult women impacted by conflict. Recent work with refugee women along the Thai-Burma border documented IPV to be a more robust predictor of suicide ideation than experiences of violent victimization during the conflict [18]. Comparable findings documenting the important role IPV plays in women’s PTSD symptoms and depression during post-conflict periods has also been described among Liberian women [19]. Nonetheless, very few other studies to date have simultaneously examined the impacts of both IPV and armed conflict on PTSD. Such work examining different forms of traumatic exposures is needed in order to further understand how different types of violence affect PTSD in order to inform strategies to optimize mental health among conflict-affected populations.

The West African nation of Côte d’Ivoire was affected by civil conflict in the early 2000’s, often referred to as “Le Crise” (i.e. “The Crisis”) or the first Ivorian Civil War. The civilian population was targeted and affected by this violence, with reports of widespread murder, rape, persecution, and terrorization throughout the country. In addition, available estimates of IPV in Côte d’Ivoire are high, with 22.2% of partnered women aged 15–49 reporting past-year physical IPV and 4.6% reporting past-year sexual IPV [20]. Given these dual burdens in Côte d’Ivoire, increased understanding of these violent exposures during and after periods of conflict and their associations with PTSD are urgently needed to guide mental health and psychosocial programming. Thus, the overall objectives of the present analysis were to: (1) assess the association between IPV experiences and past-week probable PTSD; (2) assess the association between violent experiences during the crisis and past-week probable PTSD; and (3) assess the associations between specific types of violent exposures and past-week probable PTSD.

## Materials and Methods

### Study Design

The current investigation utilized baseline data from a randomized controlled trial, “Reduction of Gender-Based Violence against Women in Post-Conflict Côte d’Ivoire” that were collected in October 2010 (Registration Number: NCT01629472). The objective of the trial was to assess the effectiveness of the incremental benefits of adding gender-dialogue groups to economic empowerment programming on reducing violence against women. The study was led by Yale School of Public Health in collaboration with Innovations for Poverty Action and the International Rescue Committee (IRC). Twenty-four rural villages in north and northwestern Côte d’Ivoire which did not have previous experience with VSLAs were selected to participate.

All women in the villages were eligible to participate in the study if they were (over 18 years of age and had no prior participation in microfinance or village savings and loans associations (VSLA) groups. IRC field staff went to the selected villages and introduced the study to village members and those interested in participating were asked to complete informed consent. Language-matched, female research staff were trained on research methodology and ethics including confidentiality, referring participants to counseling services if needed, in accordance with the World Health Organization recommendations on violence against women research [21]. Paper-based surveys were translated into French and were verbally translated by the research team during administration into eleven different local languages. The baseline survey response rate was 96% (N = 1,273) and of these, over 77% (N = 981) reported having a male partner at the time of the survey and thus were eligible for analyses pertaining to IPV. Further study details are described elsewhere [22]. All study protocols were approved by Yale School of Public Health and Innovations for Poverty Action human subjects committees.

### Measures

A 16-item post-traumatic symptom subscale of the Harvard Trauma Questionnaire (HTQ) was utilized to assess past-week post-traumatic stress disorder, as defined by the Diagnostic and Statistical Manual of Mental Disorders, 3^rd^ edition, revised (DSM-III-R) criteria [23]. The HTQ has previously been shown to have good psychometric properties in other West African and French-speaking populations [24]. Following standard procedure, all items were measured on a four-point (1–4) Likert scale and average scores of 2.0 or higher (from a theoretical range of 16–64 divided by 16) were operationalized as experiencing probable PTSD in the final binary outcome measure. The measure demonstrated high internal consistency reliability (Cronbach’s α = 0.86). We use the term probable PTSD as the measures have not been validated using the gold-standard structured diagnostic interview in our population. Violent experiences during the crisis were captured in six binary items that had previously been developed for use in Côte d’Ivoire [25] and are listed in Table 1. Any affirmative response was coded as experiencing any violence during the crisis in a summary item. Items were further categorized into binary, summary measures of crisis violence against family members, crisis violence against the woman (survey respondent), and being forced to flee.

**Table 1:** Items used to assess violence experiences during the crisis.

Variables	Items
Crisis violence against familymembers	*During or after the country’s crisis, was someone in your family was threatened;*
	*During or after the country’s crisis, was a member of your family, or someone close to you, was seriously injured or killed by an act of violence*
Crisis violence against thewoman	*During or after the country’s crisis, you were seriously injured due to a knife, gunshot, or fighting by an act of violence;*
	*During or after the country’s crisis, you were compelled to engage in sex in order to receive something such as food, protection for your family, shelter, or to cross a security check point;*
	*During or after the country’s crisis, you were forced to sleep with someone who was not your partner*
Forced to flee	*During or after the country’s crisis, you were forced to flee your village*

To examine both proximal and distal experiences of IPV which may impact mental health, a three-level categorical variable was generated to determine experiences of physical and/or sexual IPV: remote IPV (occurring within the lifetime, but not within the past twelve months), past-year IPV, and never reporting IPV. Physical and/or sexual IPV were captured via seven items from the World Health Organization’s Multi-country Study on Domestic Violence and Women’s Health [26]. The scale demonstrated acceptable reliability for remote (Cronbach’s α = 0.75 ) and past-year (Cronbach’s α = 0.75) measures. A ‘yes’ response to any item was coded as experiencing IPV in the final categorical, summary measure. Women were specifically asked if their partner ever: (1) slapped, hit, or thrown something at you; (2) pushed, shoved, kicked, or dragged you; (3) choked or burned you intentionally; (4) threatened to use a gun, knife, or other weapon against you; (5) used a gun, knife, or other weapon against you; (6) forced to have sex through threats or intimidation; and (7) physically forced to have sex when you did not want to. Demographics assessed in the analysis include age (continuous) and categorical measures of educational attainment (none versus primary, secondary or higher), religion (Christian, Muslim, Traditional, Other/None), current marital status (married versus partnered, unmarried), and ethnicity (Yacouba versus other).

### Analysis

Descriptive statistics were generated to examine the frequencies of variables among women with complete data on variables included in the analysis (n = 950; 96.8% of partnered women). To assess the relationships of interest, we utilized unadjusted logistic generalized estimating equations, which were selected to account for village-level clustering and utilized an independence correlation structure. A series of adjusted models which included one violence exposure (e.g. categorical remote or past-year IPV or a crisis violence variable) and demographic characteristics, outlined above, were then specified. To assess the differential associations between victimization exposures, remote and past-year IPV variables were added to a separate model which included crisis-related violence exposures and demographic variables in the final adjusted models. Effect modifications between exposures were assessed through interaction terms, but were not statistically significant and, therefore, were not included in the final models. Stratified models were then constructed that provided further evidence of no moderating associations between exposures. Number of types of exposures were also summed to assess dose-response relationship, but were not statistically significant and thus, not presented. Statistical significance was set at the p<0.05 level and all analyses were conducted in SAS 9.3.

## Results

### Study Characteristics

The mean age of respondents was 37.4 years (SD: 11.4) and the majority had less than a primary school educational attainment level (71.4%) (Table 2). Over 40% were Christian and 84.2% were married. Approximately 60% of women were of Yacouba ethnicity.

**Table 2:** Sample characteristics and frequency by violence exposures during the crisis and intimate partner violence (IPV) (n = 950).

Sample Characteristics		Overall^a^ % (n) or Mean (SD)	Remote IPV^b^	Past-Year IPV^b^	Any crisis violence^b^	Crisis violence against family members^b^	Crisis violence against the woman^b^	Forced to Flee^b^
Overall			26.5% (252)	23.4% (222)	72.6% (690)	48.4% (460)	7.6% (72)	56.7% (539)
Age		37.4 (11.4)	39.0 (10.3)	34.5 (10.4)	38.4 (11.2)	38.5 (10.9)	39.0 (10.5)	38.8 (11.6)
Education								
	None	71.4% (678)	25.7% (174)	21.1% (143)	71.1% (482)	45.1% (306)	6.6% (45)	57.2% (388)
	Primary, Secondary or Higher	28.6% (272)	28.9% (78)	29.0% (79)	76.5% (208)	56.6% (154)	9.9% (27)	55.5% (151)
Religion								
	Christian	43.4% (412)	27.7% (114)	20.9% (86)	74.3% (306)	49.3% (203)	7.3% (30)	58.3% (240)
	Muslim	17.2% (163)	27.6% (45)	17.8% (29)	57.7% (94)	42.9% (70)	4.9% (8)	36.8% (60)
	Traditional	16.4% (156)	21.2% (33)	30.1% (47)	75.6% (118)	53.9% (84)	9.0% (14)	59.0% (92)
	Other/None	23.1% (219)	27.4% (60)	27.4% (60)	78.5% (172)	47.0% (103)	9.1% (20)	67.1% (147)
Marital Status								
	Married	84.2% (800)	26.1% (209)	23.1% (185)	72.1% (577)	47.1% (377)	7.9% (63)	56.8% (454)
	Partnered, unmarried	15.8% (150)	28.7% (43)	24.7% (37)	75.3% (113)	55.3% (83)	6.0% (9)	56.7% (85)
Ethnicity								
	Yacouba	61.7% (586)	25.8% (151)	24.7% (145)	85.6% (502)	52.9% (310)	8.7% (51)	77.5% (454)
	Other	38.3% (364)	27.8% (101)	21.2% (77)	51.7% (188)	41.2% (150)	5.8% (21)	23.4% (85)

a Column percentages.

b Row percentages.

### Frequency of Violent Experiences during Crisis and Intimate Partner Violence

Overall, nearly three quarters (72.6%) of women reported experiencing any form of violence during the crisis. Slightly over half (56.7%) of all women reported being displaced, and nearly half (48.4%) of all women reported crisis violence against family members. Fewer women (7.6%) reported a direct personal experience of violence during the crisis (Table 2). Over a quarter (26.2%) reported lifetime IPV and over one in five (23.4%) reported IPV in the past-year. Among women reporting any remote or past-year IPV (n = 474), the most common forms of victimization were being slapped, hit, or had something thrown at them (78.7%; n = 373) and being shoved, kicked, or dragged (71.5%; n = 339). Over 40% of women (n = 196) reporting any remote or past-year violence reported having been physically forced to have sex by their partner. Having a gun, knife, or other weapon used against them was the least commonly reported form of victimization (3.4%; n = 16).

### Frequency of Probable PTSD and Bivariate Associations

In this sample, 13.1% of women met the defined cut-off for past-week probable PTSD (Table 3). In unadjusted associations, compared to women who did not report any IPV, women reporting remote IPV were found to have a 1.4 higher odds of probable PTSD (95%CI: 0.8–2.3 p = 0.2); though this association did not reach statistical significancePast-year IPV was significantly associated with increased odds of probable PTSD (OR: 2.8; 95%CI: 1.7–4.5; p<0.001). While increased likelihood of probable PTSD was observed among women who reported crisis-related violence (summary measure as well as specific forms), none of the bivariate associations reached statistical significance. Older age (OR: 1.03; 95%CI: 1.005–1.05; p = 0.01), educational attainment of primary, secondary, or higher (OR: 0.5; 95%CI: 0.3–0.8; p = 0.005), traditional religion (OR:5.5; 95%: 2.8–11.1; p<0.001) and other ethnicities (OR: 0.2; 95%CI: 0.1–0.3; p<0.001) were significantly associated with probable PTSD. No other demographics were significantly associated with probable PTSD.

**Table 3:** Frequency of past-week probable PTSD and bivariate associations with traumatic exposures and demographics (n = 950).

		Probable PTSD	Unadjusted Odds^a^
		% (N) or Mean (SD)	OR (95% CI)
Overall		13.1% (124)	–
	**Violence Exposures**		
IPV			
	None	9.2% (44)	Ref
	Remote IPV	12.3% (31)	1.4 (0.8–2.3)
	Past Year IPV	22.1% (49)	2.8 (1.7–4.5)***
Any Crisis Violence			
	Yes	14.5% (100)	1.6 (0.9–2.7)
	No	9.2% (24)	
Crisis violence against family members			
	Yes	14.6% (67)	1.3 (0.9–1.8)
	No	11.6% (57)	
Crisis violence against the woman			
	Yes	20.8% (15)	1.7 (0.9–3.0)
	No	12.4% (109)	
Forced to Flee			
	Yes	15.8% (85)	1.7 (0.9–3.1)
	No	9.5% (39)	
	**Demographics**		
Age		40.3 (9.6)	1.03 (1.005–1.05)**
Education			
	None	15.2% (103)	Ref
	Primary, Secondary or Higher	7.7% (21)	0.5 (0.3–0.8)**
Religion			
	Christian	8.5% (35)	Ref
	Muslim	4.3% (7)	0.5 (0.2–1.3)
	Traditional	34.0% (53)	5.5 (2.8–11.1)***
	Other/None	13.2% (29)	1.6 (0.9–3.1)
Marital Status			
	Married	13.3% (106)	Ref
	Partnered, unmarried	12.0% (18)	0.9 (0.5–1.7)
Ethnicity			
	Yacouba	18.6% (109)	Ref
	Other	4.1% (15)	0.4 (0.3–0.6)***

a Two women not included in models due to missing data on village.

*p<0.05, **p<0.01, ***p<0.001.

### Adjusted Associations with Probable PTSD

Accounting for demographics (age, education, religion, marital status, and ethnicity), women who reported remote IPV had a 1.6 higher odds of past-week probable PTSD symptoms in comparison to those who did not report IPV experiences (95%CI: 0.9–2.6; p = 0.09) (Table 4). This relationship was not observed to reach statistical significance. The association between past-year IPV and probable PTSD was observed to have a more robust relationship; women who reported past-year IPV had 3.1 times the odds of reporting probable PTSD compared to women who did not report any IPV (95%CI: 1.8–5.3; p<0.001). After inclusion of violence during crisis experiences, as well as demographics, the association between past-year IPV and past-week probable PTSD was not attenuated, while remote IPV remained non-significant. Experiences of any violence during crisis nor was not significantly associated with past week probable PTSD. The associations remained non-significant and did not attenuate after including IPV variables in the models.

**Table 4:** Adjusted odds ratios of violence exposures and past-week probable PTSD.

		Adjusted Odds Ratios for Probable PTSD^c^							
		aOR (95%CI)^a^	aOR (95%CI)^a^	aOR (95%CI)^a^	aOR (95%CI)^a^	aOR (95%CI)^a^	aOR (95%CI)^a^	aOR (95%CI)^a^^,^ b	aOR (95%CI)^a^^,^ b
	**Violence Exposures**								
IPV								2.2 (1.4–3.6)**	
	None	Ref							
	Remote IPV	1.6 (0.9–2.6)							2.6 (1.6–4.1)***
	Past Year IPV		3.1 (1.8–5.3)***						
Any Crisis Violence				1.04 (0.7–1.5)					
Crisis violence against family members					1.1 (0.8–1.7)			1.02 (0.7–1.5)	1.1 (0.8–1.6)
Crisis violence against the woman						1.7 (0.7–3.7)		1.7 (0.8–3.6)	1.5 (0.7–3.4)
Forced to Flee							0.9 (0.5–1.7)	0.9 (0.6–1.7)	0.9 (0.5–1.7)
	**Demographics**								
Age		1.01 (0.9–1.03)	1.01 (0.9–1.03)	1.01(0.9–1.03)	1.01 (0.9–1.03)	1.02 (0.9–1.04)	1.02 (0.9–1.04)	1.02 (0.9–1.04)	1.0 (0.9–1.04)
Education									
	None	Ref	Ref	Ref	Ref	Ref	Ref	Ref	Ref
	Primary, Secondary or Higher	0.6 (0.4–1.1)	0.6 (0.4–1.1)	0.6 (0.4–1.1)	0.6 (0.4–1.2)	0.6 (0.3–1.05)	0.6 (0.3–1.02)	0.6 (0.3 -–1.01)	0.6 (0.3–0.9)*
Religion									
	Christian	Ref	Ref	Ref	Ref	Ref	Ref	Ref	Ref
	Muslim	0.7 (0.2–2.3)	0.7 (0.2–2.2)	0.7(0.2–2.3)	0.7 (0.2–2.2)	0.7 (0.2–2.4)	0.7 (0.2–2.5)	0.7 (0.2–2.4)	0.7 (0.2–2.4)
	Traditional	4.4 (2.3–8.5)***	4.4 (2.3–8.5)***	4.4(2.3–8.4)***	4.4 (2.3–8.4)***	4.4 (2.3–8.2)***	4.1 (2.1–7.7)***	4.4 (2.4–8.0)***	4.0 (2.1–7.5)***
	Other/None	1.3 (0.7–2.4)	1.3 (0.7–2.4)	1.3(0.7–2.4)	1.3 (0.7–2.4)	1.3 (0.7–2.3)	1.3 (0.7–2.3)	1.3 (0.7–2.2)	1.2 (0.7–2.2)
Marital Status									
	Married	Ref	Ref	Ref	Ref	Ref	Ref	Ref	Ref
	Partnered, unmarried	1.2 (0.6–2.2)	1.1 (0.6–2.1)	1.2(0.6–2.2)	1.2 (0.6–2.2)	1.1 (0.6–2.1)	1.2 (0.6–2.2)	1.2 (0.6–2.2)	1.2 (0.6–2.2)
Ethnicity									
	Yacouba	Ref	Ref	Ref	Ref	Ref	Ref	Ref	Ref
	Other	0.5 (0.4–0.7) ***	0.5 (0.4–0.7)***	0.5 (0.4–0.7)***	0.5 (0.4–0.7)***	0.3 (0.1–0.9)***	0.5 (0.4–0.7)***	0.5 (0.4–0.7)***	0.5 (0.4–0.7)*
QICu Fit Criteria^c^		652.2	651.8	650.1	652.2	637.9	634.4	641.7	638.8

a Odds ratios adjusted age, education, ethnicity, religion, and marital status.

b Adjusted for other exposures in column.

c Quasi-likelihood under the Independence model Criterion: Fit statistic for comparing generalized estimating equation (GEE) models.

Note:Two women not included in models due to missing data on village.

*p<0.05, **p<0.01, ***p<0.001.

### Post-Hoc Analyses

To further examine the associations between the type of IPV and relations with probable PTSD, additional post-hoc analyses were conducted. Such analyses revealed that women who reported severe IPV (i.e. any sexual violence, choked, burned intentionally, threatened or used a gun, knife, or other weapon) at some point in their lifetime had heightened odds of probable PTSD compared to women who did not report severe IPV (OR: 2.9; 95%CI: 1.9–4.5; p<0.001), after accounting for demographics.

## Discussion

This study with rural Ivorian women documented a high burden of current probable PTSD, with past year experiences of IPV being associated with increased odds of poor mental health. Importantly, in adjusted models, past-year IPV experiences was a more stronger and more consistent correlate of current probable PTSD than violence experienced during the crisis. These findings underscore the importance of considering this very private form of violence as a critical determinant of mental well-being in this West African setting. Our findings of past-year IPV being a more robust predictor of poor mental health than war-related violence is also consistent with the growing body of research documenting the harmful impacts of daily forms of suffering on the mental health of conflict-affected populations [18], [27], [28]. Taken together, study findings from diverse countries and populations lend further support to the importance of broadening current conceptions of suffering in war-affected settings to also include violence exposures, such as physical and sexual IPV that are not necessarily perpetrated by armed actors or otherwise direct consequences of a war [29].

Additional research is needed to clarify mechanisms for observed associations. Certainly, as shown in existing work, armed conflict exposure is detrimental to mental health [30]. However, more understanding is needed as to why IPV was more predictive of probable PTSD above and beyond that of armed conflict exposure in this population. The current study does not allow us to examine what specific aspects of these traumatic violence exposures may be most relevant to increasing risk for PTSD. However, the post-hoc finding that severe IPV strongly predicts probable PTSD may indicate that more severe types of partner-perpetrated trauma may have more harmful, lasting impacts on women’s mental health; more research is needed. Exposure to armed violence during the conflict may have been an indirect and/or one-time occurrence, while IPV is directly experienced and may either be chronic or acute. The increased odds for probable PTSD may be related to IPV serving as a proxy measure for a broader range of family dysfunction and multiple unmeasured daily stressful life events in this population. It is possible that women are better able to compartmentalize their past experiences of conflict violence, compared with their current domestic life of pervasive violence and strife. Furthermore, as discussed with other types of violence (e.g. child abuse from close relatives/trusted acquaintances), perpetration of violence by an intimate partner may be more psychologically traumatic than by a stranger in a conflict setting [31]–[34]. In addition, the recency of the traumatic events may differ between these two exposure types, as the armed conflict under study occurred almost a decade prior to data collection, thus potentially affecting the association with current PTSD. Current findings also suggest the importance of the timing of victimization perpetrated by partners may also be important in understanding women’s current mental health and suggests that programs should target efforts to women who have recently experienced IPV victimization. Further research is needed.

The lack of programmatic attention to IPV in conflict-affected settings may also contribute to the findings reported herein. Prior research and reports from humanitarian organizations indicate that IPV is often under-prioritized in conflict-affected settings [10], [35]. Thus, services (e.g. counseling, support groups) that are needed to mitigate the harmful health impacts, including mental health, are scarce. Similarly, while war-related violence may be more openly discussed since most community members are exposed (either directly or indirectly), IPV is widely perceived as a private matter. Two community-based survey reports found that 40–50% of women with IPV experiences did not disclose their IPV experience to anyone, including family and friends [25], [36]. Such difficulty with obtaining support from others may in turn compromise women’s mental health [37]–[39].

Future research, including longitudinal assessments combined with qualitative work, are needed to better understand the observed associations and possible mechanisms, This may be particularly challenging due to contention regarding the best way to capture the effects of multiple potentially traumatic exposures and account for their inter-correlation and potential causal associations [40], [41]. This is especially true of post-conflict societies where the entire community has been traumatized and continues to struggle with the difficulties of daily living [42]. Future work should also seek to better understand protective factors that may buffer the impact of IPV among conflict-affected women. Our findings also show that ethnicity and religion are statistically associated with current probable PTSD in this sample. Research should explore these factors as possible effect modifiers for violence exposure, and conduct multi-level analyses to assess the compositional effects of ethnicity and religion on different groups, with respect to exposure to conflict and interpersonal trauma.

The findings of this study must be interpreted within the context of important limitations. Firstly, the HTQ has not been validated in Côte d’Ivoire. Similarly, assessments of local perceptions of poor mental health were not conducted, and there is limited research on post-traumatic symptomology in this population. Additionally, study participants are drawn from a community-based sample as part of a larger intervention study. Thus, findings can only be generalized to the women whose demographics are represented in this study. As with other stigmatized health behaviors and experiences, women may have under-reported on their experiences with both IPV and PTSD. Such under-reporting would likely only impact the overall statistical power as opposed to directionality of the association, unless women with probable PTSD were more likely to over- or under-report their exposure to violence. Due to potentially greater relative acceptance of more public forms of violence exposures, such as being forced to flee, there is a possibility that the threat of under-reporting was less for violence during conflict than for IPV experiences. Despite this possibility of differential under-reporting, IPV remained a stronger predictor of probable PTSD than war-related violence. We were also unable to assess other forms of trauma, violence, neglect, or hardship that women may have experienced in the past and that may also confound the associations observed herein. Future research should attempt to collect a more thorough range of lifetime potentially traumatic experiences from conflict-affected populations. Lastly, the cross-sectional nature of the data precludes the determination of causal relationships. However, the threat of reverse causation is partly reduced in that experiences of conflict violence and IPV were likely temporally prior to the experience of past-week probable PTSD.

These limitations notwithstanding, findings from this study with rural Ivorian women highlight important implications for mental health and humanitarian programming. Firstly, efforts to address the impacts of everyday suffering, especially past-year IPV, must be integrated into existing programs in conflict-affected settings. This may include culturally-appropriate mental health services to address the needs of women who suffer from poor mental health and who are at continued risk of IPV. Given the potential reluctance of women to report IPV [25], [36], future research can seek to investigate the feasibility and impact of integrating such services into open women’s groups. Secondly, initiatives to prevent and/or reduce ongoing IPV must be prioritized in regions impacted by conflict. This may include mobilizing communities to change gender norms disfavoring women, outreach to men and boys, and strengthening response services for IPV. Structural interventions, including initiatives that seek to integrate economic empowerment programming with gender training have been shown to be promising in reducing IPV [22], [43]. Therapeutic interventions may also be important as at least one intervention study with survivors or sexual violence showed improved mental health [44]. Longitudinal epidemiologic research is also needed to understand relationships between multiple forms of violence and to identify opportunities to promote resilience. Future epidemiologic, qualitative, and intervention research with communities impacted by conflict that integrate the impacts of IPV on mental health will have the potential to improve the health and well-being of this critically under-served population.
